# Therapeutic Effects of PPARα Agonist on Ocular Neovascularization in Models Recapitulating Neovascular Age-Related Macular Degeneration

**DOI:** 10.1167/iovs.17-22091

**Published:** 2017-10

**Authors:** Fangfang Qiu, Greg Matlock, Qian Chen, Kelu Zhou, Yanhong Du, Xiang Wang, Jian-Xing Ma

**Affiliations:** 1 Department of Physiology, The University of Oklahoma Health Sciences Center, Oklahoma City, Oklahoma, United States; 2 Department of Cell Biology, The University of Oklahoma Health Sciences Center, Oklahoma City, Oklahoma, United States

**Keywords:** age-related macular degeneration, PPARα, fenofibrate, inflammation, retina, neovascularization

## Abstract

**Purpose:**

This study was designed to evaluate effects of fenofibric acid (Feno-FA), a peroxisome proliferator–activated receptor-alpha (PPARα) agonist, on ocular neovascularization (NV) in models recapitulating neovascular age-related macular degeneration (AMD), and to explore whether the effects are PPARα dependent.

**Methods:**

Laser-induced choroidal NV (CNV) in rats and very low-density lipoprotein receptor knockout (*Vldlr^−/−^*) mice received daily intraperitoneal injections of Feno-FA or vehicle. Vascular leakage was examined by fundus fluorescein angiography and permeability assay using Evans blue as tracer. In CNV rats, severity of CNV was evaluated by CNV areas and CNV volume. In *Vldlr^−/−^* mice, subretinal NV (SRNV) and intraretinal NV (IRNV) were quantified in choroid flat mount and retina flat mount, respectively. Inflammatory factors were measured using Western blotting and retinal leukostasis assay. Further, *Pparα^−/−^* mice and age-matched wild-type (WT) mice were used for laser-induced CNV and treated with Feno-FA to explore the underlying mechanism.

**Results:**

Feno-FA significantly reduced vascular leakage in CNV rats and *Vldlr^−/−^* mice, reduced CNV volume in laser-induced CNV rats, and suppressed SRNV and IRNV in *Vldlr^−/−^* mice. In addition, Feno-FA downregulated the expression of inflammatory factors, including VEGF, TNF-α, and intercellular cell adhesion molecule-1 (ICAM-1), in the eyecups of CNV rats and decreased adherent retinal leukocytes in *Vldlr^−/−^* mice. Furthermore, *Pparα^−/−^* mice developed more severe CNV compared with WT mice, and PPARα knockout abolished the beneficial effects of Feno-FA on CNV.

**Conclusions:**

Feno-FA has therapeutic effects on ocular NV in models recapitulating neovascular AMD through a PPARα-dependent mechanism.

Age-related macular degeneration (AMD) is the major cause of permanent visual damage in the elderly global population.^1–3^ There are two forms of AMD: atrophic AMD and neovascular AMD. Neovascular AMD, responsible for vision loss in approximately 90% of AMD patients, is characterized by choroidal neovascularization (CNV), which refers to the aberrant sprouting of new, immature blood vessels into the subretinal space accompanied by inflammation, vascular leakage, retinal edema, and vision loss.^1,4,5^ However, there are no satisfactory noninvasive treatments for this ocular disorder currently. Hence, there is an urgent need to develop novel systemic drug treatment strategies for this disease.

Peroxisome proliferator–activated receptor α (PPARα), a member of nuclear receptor superfamily, is a ligand-activated transcription regulator expressed in multiple organs, including the retina, kidney, and liver.^6–8^ PPARα activation requires binding of ligands,^9^ or therapeutic synthetic fibrate drugs such as fenofibrate.^10,11^ PPARα was originally recognized for its hypolipidemic effects. PPARα activation is also associated with anti-inflammatory and antiangiogenic properties.^12–16^ Fenofibrate, which is converted to fenofibric acid (Feno-FA), a specific PPARα agonist, has been shown by two independent clinical studies, the FIELD and ACCORD studies, to have therapeutic effects on microvascular complications in patients with type 2 diabetes.^17,18^ Furthermore, our recent study demonstrated that PPARα activation by fenofibrate had beneficial effects on diabetic retinopathy (DR) in a type 1 diabetic animal model and ischemia-induced retinal NV.^19^ In addition, a recent study reported that fenofibrate inhibited laser-induced CNV.^20^ Therefore, we hypothesize that PPARα activation may have beneficial effects on neovascular AMD, which have been recently proposed.^21^

In the present study, we investigated whether PPARα activation using systemic administration of Feno-FA had beneficial effects on ocular NV in models recapitulating some features of neovascular AMD including a laser-induced CNV rat model and very low-density lipoprotein receptor knockout (*Vldlr^−/−^*) mice, a genetic subretinal NV (SRNV) and intraretinal NV (IRNV) model. Further, we have explored the underlying mechanism.

## Materials and Methods

### Animals

Male Brown Norway rats (8–10 weeks old; Charles River, Wilmington, MA, USA), *Vldlr^−/−^* mice (postnatal day [P]13–P28), *Pparα^−/−^* mice (8–10 weeks old), and wild-type (WT) C57BL/6J mice (8–10 weeks old; Jackson Laboratories, Bar Harbor, ME, USA) were used. Breeding pairs of *Vldlr^−/−^* mice and *Pparα^−/−^* mice are in the background of C57BL/6J, purchased from Jackson Laboratories. All experiments were performed following the guidelines of the ARVO Statement for the Use of Animals in Ophthalmic and Vision Research and approved by the Institutional Animal Care and Use Committee of the University of Oklahoma Health Sciences Center. In all procedures, animals were anesthetized with intramuscular injection of 50 mg/kg ketamine hydrochloride mixed with 5 mg/kg xylazine (Vedco, St. Joseph, MO, USA), and pupils were dilated with topical administration of 1% cyclopentolate (Wilson, Mustang, OK, USA).

### Laser-Induced CNV

CNV was induced by laser in Brown Norway rats, *Pparα^−/−^*, and WT mice as described previously.^22^ Briefly, photocoagulation was performed in anesthetized animals with dilated pupils using a laser photocoagulator (532-nm wavelength; model diode pumped solid-state; Ellex Medical PTY, Adelaide, Australia). The parameters were as follows: spot size, 75 μm; duration, 100 ms; power, 500 mW for rats, 200 mW for mice. Four spots for mice or eight spots for rats were applied to each eye between the major retinal vessels around the optic disc at a distance of approximately 2 optic disc diameters from the optic nerve head. Only burns that generated a bubble were included in the study.

### Intraperitoneal Injection of Feno-FA

Feno-FA (AK Scientific, Union, CA, USA) was prepared in dimethyl sulfoxide (DMSO). Animals were intraperitoneally injected daily with Feno-FA (25 mg/mL) or vehicle (DMSO) at a volume of 1 μL/g body weight. Treatment was given to rats for 2 weeks, *Pparα^−/−^* and WT mice for 1 week, beginning at the same day as the laser photocoagulation to the day of analysis, and to *Vldlr^−/−^* mice from P13 to P28.

### Optical Coherence Tomography (OCT) and Quantification of CNV Volume

Spectral-domain (SD) OCT was performed using the SD-OCT device (Bioptigen, Inc., Durham. NC, USA) as described previously.^23^ Images were captured using the following parameters: rectangular scan: 1000 A-scans per B-scan, 100 B-scans per frame. Total retinal thicknesses were measured perpendicular to the surface of the RPE layer and 500 μm away from the center of the optic nerve at 12, 3, 6, and 9 o'clock direction with built-in software. All B-scan sections crossing the CNV were selected for analysis. CNV volume (μm^3^) was calculated with the following formula: Σ*_n_*(*a_n_*·*t_n_*) (in which *a_n_* is the area and *t_n_* the thickness of the *n*th B-scan). The area of each B-scan passing through a CNV was calculated using ImageJ software (http://imagej.nih.gov/ij/; provided in the public domain by the National Institutes of Health, Bethesda, MD, USA).

### Fundus Fluorescein Angiography (FFA)

FFA was performed using the Micron III fundoscopy system (Phoenix Research Labs, Pleasanton, CA, USA) as described previously.^24^ Briefly, anesthetized animals with dilated pupils were intraperitoneally injected with 5% sodium fluorescein (Akorn, Decatur, IL, USA; 100 μL/mouse and 1 mL/rat). Images were captured at predesignated times (1, 3, 5, and 7 minutes). For CNV rats, fluorescein leakage was graded as described previously^22^: grade 0: no leakage, faint hyperfluorescence, or speckled fluorescence without leakage; grade 1: questionable leakage, hyperfluorescent lesion without advancing increase in size or intensity; grade 2: leaky, hyperfluorescence increasing in intensity but not significantly in size without definite leakage; grade 3: pathologically significant leakage, hyperfluorescence increasing in intensity and in size with definite leakage. Only lesions with a leakage of grade 3 were considered clinically significant. For *Vldlr^−/−^* mice, the numbers of fluorescein leakage spots at 3 minutes after injection were used for analysis.

### Choroidal Flat Mount and Retina Flat Mount Following Angiography Using Fluorescein Isothiocyanate–Conjugated Dextran (FITC-D)

The anesthetized animals were perfused with FITC-D (2 × 10^6^ molecular weight; 20 mg/mL; Sigma-Aldrich Corp., St. Louis, MO, USA) through the femoral vein. The eyes were enucleated and fixed in 4% paraformaldehyde for 2 hours. The retina and eyecup including the RPE, choroid, and sclera were flat-mounted separately in a mounting medium (Richard Allan Scientific, Kalamazoo, MI, USA) on slides. The images were captured using fluorescence microscopy. The total numbers of NV were counted and NV areas were measured using ImageJ software.

### Retinal Vascular Permeability Assay

As described previously,^25^ Evans blue dye (Sigma-Aldrich Corp.) was injected into anesthetized animals through the femoral vein (30 mg/kg body weight). After 2 hours, Evans blue dye in the circulation was removed by perfusion with 0.1M citrate buffer with 1% paraformaldehyde (pH 4.2). The retina was homogenized and Evans blue was extracted. Concentrations of Evans blue from the supernatant were measured with a spectrophotometer (DU800; Beckman Coulter, Brea, CA, USA) and normalized by total retinal protein concentration.

### Retinal Leukostasis Assay

The assay was performed as described previously.^19^ Briefly, anesthetized mice were perfused with PBS to remove nonadherent leukocytes, and the adherent leukocytes in the vasculature were stained by perfusion with FITC-conjugated concanavalin-A (200 μg/mL). The retina was flat-mounted, and adherent leukocytes in the artery, vein, and their first-grade branches were counted under a fluorescence microscope.

### Western Blot Analysis

The eyecup was homogenized and lysed in RIPA buffer. The equal amount (50 μg/lane) of total proteins was resolved by SDS polyacrylamide gel electrophoresis and electrotransferred onto a nitrocellulose membrane. The membrane was blocked with 5% nonfat milk for 1 hour, followed by incubation with a primary antibody overnight at 4°C and subsequently the secondary antibody for 1 hour. The signal was developed using enhanced chemiluminescence reagents (Pierce, Rockford, IL, USA) and quantified by densitometry using ImageJ and normalized by β-actin levels. Primary antibody dilutions were 1:1000 for the rabbit anti-ICAM-1 antibody (catalog number: ab27536) and rabbit anti-TNF-α antibody (ab9739-100), 1:500 for rabbit anti-VEGF antibody (ab46154), which were purchased from Abcam (Cambridge, MA, USA), and 1:5000 for mouse anti-β-actin antibody (catalog number: A5441, Sigma-Aldrich Corp.).

### Statistical Analysis

Statistical analysis was performed using SPSS 15.0 software (Chicago, IL, USA). Data were expressed as % or mean ± standard error (SEM). A categorical variable was compared using the *χ*^2^ test. Quantitative data were analyzed using unpaired Student's *t*-test for comparison between two groups and 2-way analysis of variance (ANOVA) followed by Tukey's post hoc tests for comparison among three or more groups. *P* < 0.05 was considered statistically significant.

## Results

### Activation of PPARα Reduces Vascular Leakage From Laser-Induced CNV

**Figure 1:**
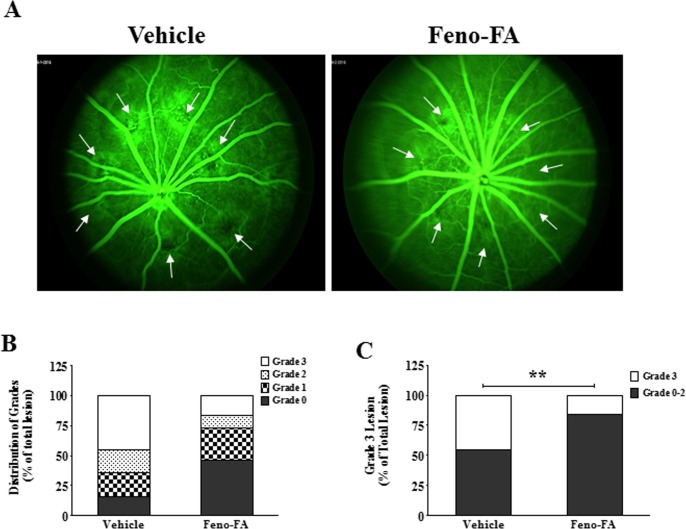
Fenofibric acid (Feno-FA) reduced vascular leakage in the laser-induced choroidal neovascularization (CNV) model. Brown Norway rats were intraperitoneally injected daily with Feno-FA or an equal volume of vehicle, beginning at the same day as the laser treatment. Fluorescein fundus angiography (FFA) was performed at 2 weeks after laser photocoagulation. (A) Representative images of FFA leakage in the vehicle group and Feno-FA group. White arrows indicate the CNV lesions. (B) The percentages of grade 0, 1, 2, and 3 lesions were calculated in the vehicle group (n = 35) and Feno-FA group (n = 37). (C) The percentages of grade 3 CNV lesions were quantified and compared between the vehicle and Feno-FA groups. Data were analyzed by χ^2^ test. **P < 0.01.

We first determined whether PPARα activation reduces vascular leakage from laser-induced CNV in rats. As shown by FFA in Figure 1, the incidence of grade 3 lesions, clinically significant CNV lesions, was 16.2% in the Feno-FA group (*n* = 37) and 45.2% in the vehicle group (*n* = 35), indicating a decrease of 64.2% in grade 3 lesion by Feno-FA. This result suggests an inhibitory effect of PPARα activation by Feno-FA on CNV leakage.

### Activation of PPARα Inhibits Laser-Induced CNV

**Figure 2:**
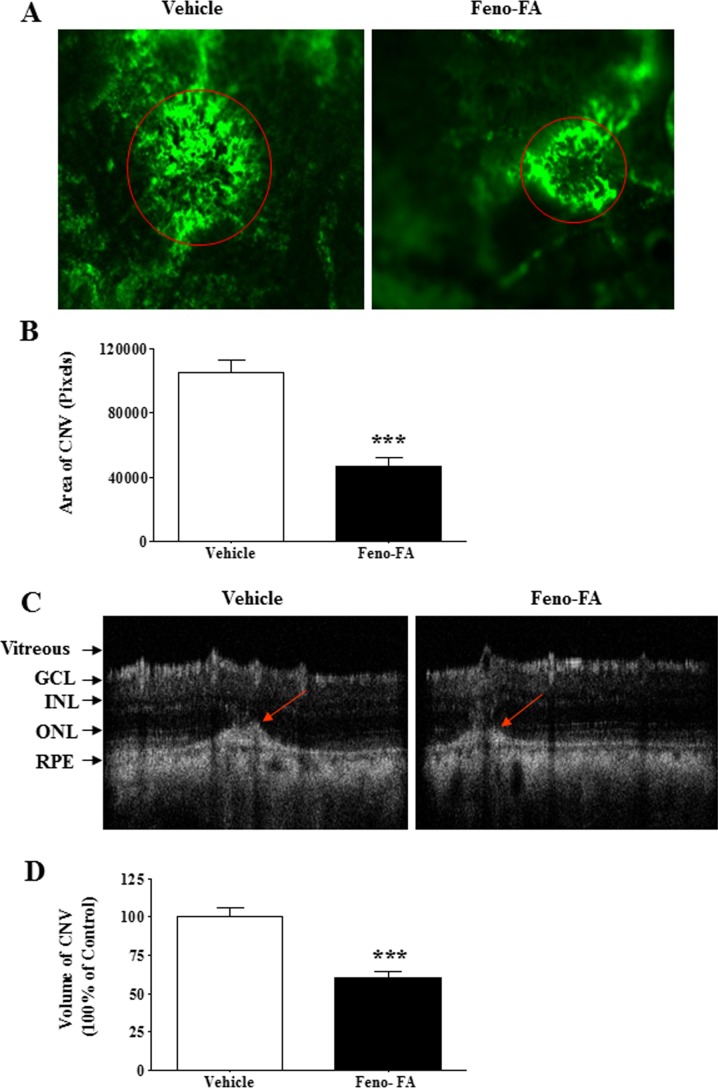
Feno-FA inhibited laser-induced CNV. Brown Norway rats were intraperitoneally injected daily with Feno-FA or an equal volume of vehicle, beginning at the same day as the laser treatment. The area of CNV was measured in flat-mounted RPE-choroidal-scleral complex perfused with FITC-dextran, and the CNV volume was measured OCT at 2 weeks after the laser treatment. (A) Representative images of CNV in the flat mount perfused with FITC-dextran; red circles indicate representative CNV lesions. (B) Quantification of CNV areas in the flat mount in the vehicle group (n = 11) and Feno-FA group (n = 16). (C) Representative CNV images in OCT; red arrows indicate CNV lesion. (D) Quantification of CNV volume on OCT images in the vehicle group (n = 23) and Feno-FA group (n = 26). Data were expressed as mean ± SEM, and analyzed by unpaired Student's t-test. ***P < 0.001.

Furthermore, we investigated whether PPARα activation inhibits laser-induced CNV. As shown by flat-mounted choroid, FITC-D CNV areas were 104,900 ± 7567 (pixels) in the vehicle group (*n* = 11) and 47,000 ± 5299 (pixels) in the Feno-FA group (*n* = 16), decreasing by 55.2% in the Feno-FA group, compared with the vehicle group (Figs. 2A, 2B). Moreover, as shown by OCT, the mean CNV volume was also decreased by 40.0% in the Feno-FA group (*n* = 26), compared with the vehicle group (*n* = 23) (Figs. 2C, 2D). These results demonstrated that PPARα activation suppresses laser-induced CNV.

### Activation of PPARα Attenuates Overexpression of Inflammatory Factors in Eyecups of Rat With Laser-Induced CNV

**Figure 3:**
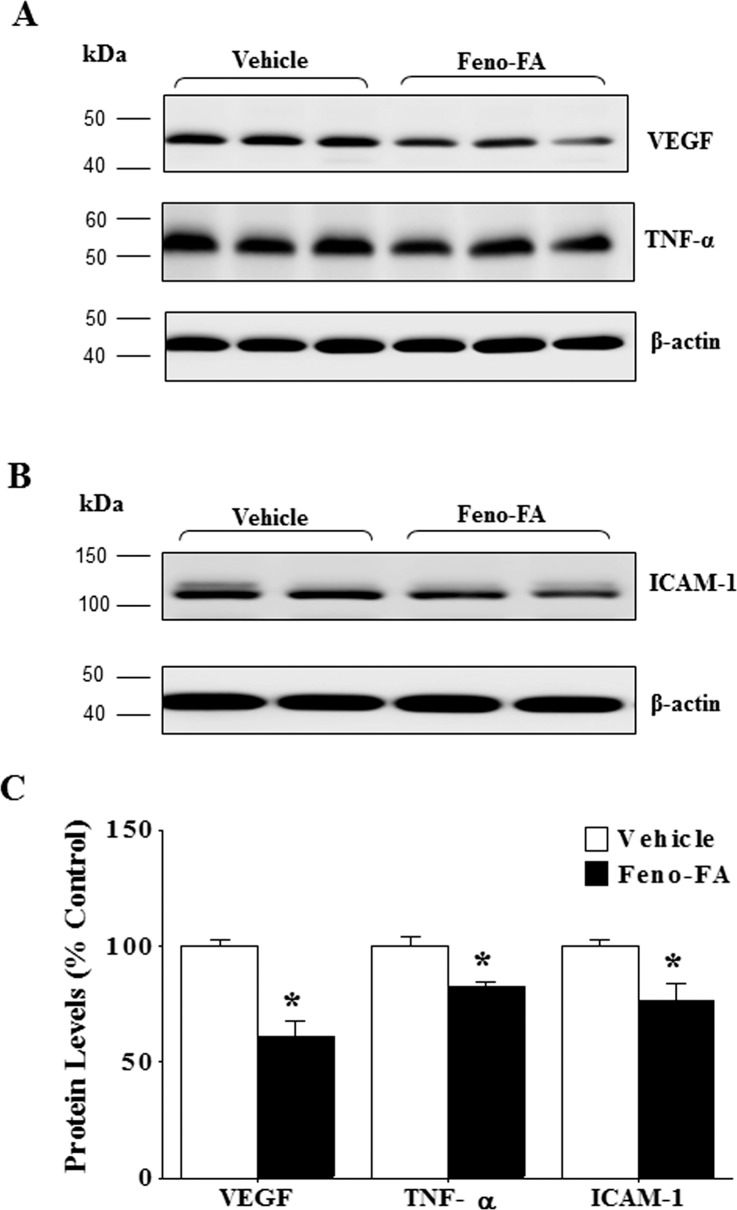
Feno-FA attenuated overexpression of inflammatory factors in rat eyecup of laser-induced CNV. Rats with laser-induced CNV were intraperitoneally injected daily with Feno-FA or an equal volume of vehicle for 2 weeks, beginning at the same day as the laser treatment. (A, B) Two weeks after laser photocoagulation, the same amount (50 μg) of eyecup proteins from each rat was used for Western blot analysis of VEGF and TNF-α (A) and ICAM-1 (B); (C) results were semiquantified by densitometry and normalized by β-actin levels. Data were expressed as percentages (%) of control and analyzed by unpaired Student's t-test; n = 3–5/group; *P < 0.05.

Inflammation plays an important role in the pathogenesis of CNV.^26,27^ Therefore, we examined the effect of PPARα activation on expression of inflammatory factors in a rat model of laser-induced CNV. As shown by Western blot analysis, Feno-FA significantly decreased protein levels of VEGF, TNF-α, and ICAM-1 in the rat eyecups with CNV (Fig. 3), compared with vehicle group, suggesting inhibitory effects of PPARα activation on inflammatory response in the rat's eyecup with laser treatment.

### Activation of PPARα Reduces Retinal Vascular Leakage in *Vldlr*^−/−^ Mice

**Figure 4:**
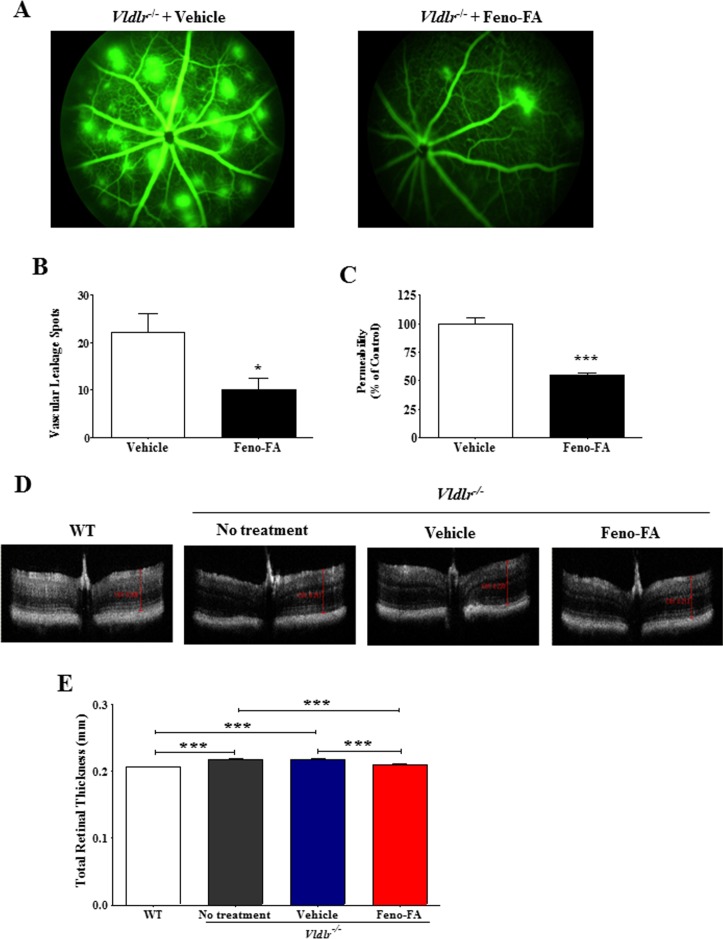
Feno-FA reduced retinal vascular leakage in very low-density lipoprotein receptor knockout (Vldlr^−/−^) mice. Vldlr^−/−^ mice were intraperitoneally injected daily with Feno-FA or vehicle (DMSO) from P13 to P28. Retinal vascular leakage was examined using FFA, permeability assay using Evans blue dye as a tracer, and total retinal thickness measured on OCT at P28. (A) Representative images of FFA in Vldlr^−/−^ mice at 3 minutes after the injection of the dye. (B) Quantification of leakage spot numbers in the vehicle and Feno-FA groups (n = 6/group). (C) Quantification of vascular permeability using Evans blue dye as tracer in the vehicle group (n = 12) and Feno-FA group (n = 11). Evans blue concentration was normalized by total retina protein concentrations. (D) Representative retinal images of OCT; (E) quantification of total retinal thickness on OCT images in age-matched wild-type (WT) mice (n = 22), Vldlr^−/−^ mice (n = 12), Vldlr^−/−^ mice treated with vehicle (n = 10), and Vldlr^−/−^ mice treated with Feno-FA (n = 10). Data were expressed as mean ± SEM. Data of vascular leakage spots and permeability were analyzed by unpaired Student's t-test; data of total retinal thickness on OCT were analyzed by 2-way analysis of variance (ANOVA) followed by Tukey's post hoc tests. *P < 0.05; ***P < 0.001.

In *Vldlr^−/−^* mice, we also evaluated the effects of PPARα activation on retinal vascular leakage. As shown by FFA, there were numerous intense hyperfluorescent spots representing dye leakage occurring throughout the entire retina in *Vldlr^−/−^* mice with vehicle treatment, while there were fewer leakage spots in those of the Feno-FA treatment group (Fig. 4A). Quantification of the results showed that Feno-FA treatment significantly decreased the number of vascular leakage spots in the retina of *Vldlr^−/−^* mice, compared to vehicle control (Fig. 4B). Our previous study showed that vascular permeability in the retina of *Vldlr^−/−^* mice was approximately 2-fold higher over that in WT mice.^22^ Here, we found that Feno-FA significantly reduced the retinal vascular permeability in *Vldlr^−/−^* by 45.6% compared with vehicle control shown by using Evans blue dye as tracer (Fig. 4C).

In addition, we measured total retinal thickness using OCT and found that it was increased in *Vldlr^−/−^* mice (0.217 ± 0.0014 mm; *n* = 12) compared to age-matched WT mice (0.206 ± 0.0008 mm; *n* = 22), suggesting retinal edema. To exclude the possibility that the increased retina thickness could be due to the increased retinal cell numbers, we further examined the retinal morphology using age-matched mice by hematoxylin and eosin staining, and found that the cell numbers were not significantly changed in various retinal layers including the outer nuclear layer, inner nuclear layer, and ganglion cell layer in *Vldlr^−/−^* mice compared with WT mice (Supplementary Fig. S1). These results indicate that the increase of total retinal thickness is likely attributed to retinal edema in *Vldlr^−/−^* mice. On the other hand, Feno-FA treatment significantly decreased retina thickness in *Vldlr^−/−^* mice (0.209 ± 0.0014; *n* = 10), compared to the vehicle group (0.218 ± 0.001; *n* = 10) (Figs. 4D, 4E), but showed no effect on the cell numbers in the various retinal layers mentioned above (Supplementary Fig. S1), suggesting that Feno-FA reduces retinal edema in *Vldlr^−/−^* mice. All in all, PPARα activation significantly reduced retina vascular leakage in *Vldlr^−/−^* mice.

### Activation of PPARα Decreases SRNV and IRNV in *Vldlr^−/−^* Mice

**Figure 5:**
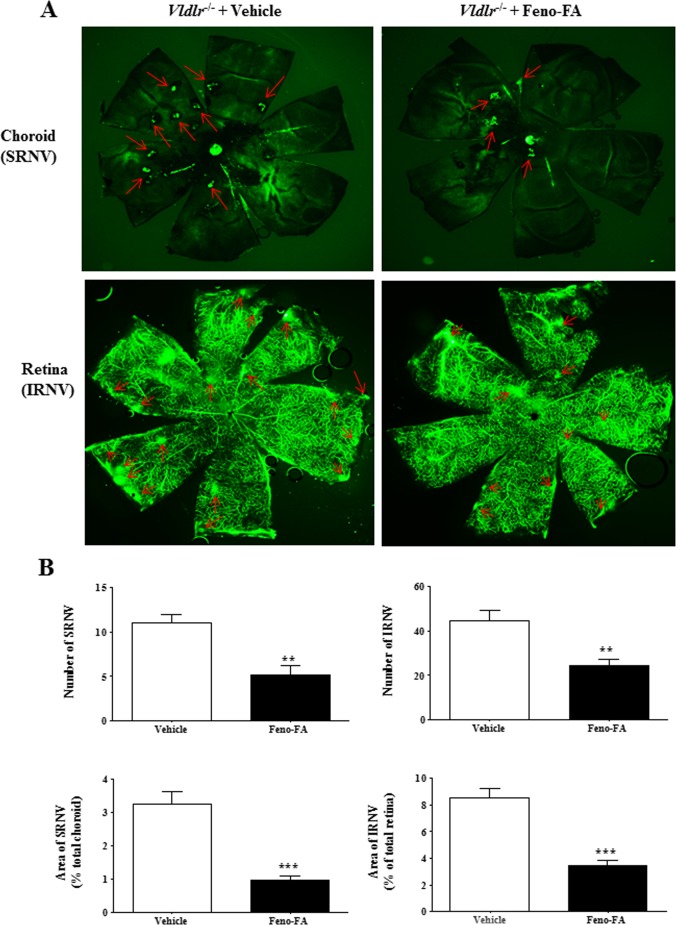
Feno-FA ameliorated subretinal neovascularization (SRNV) and intraretinal NV (IRNV) in Vldlr^−/−^ mice. Vldlr^−/−^ mice were intraperitoneally injected daily with Feno-FA or vehicle from P13 to P28. SRNV and IRNV were quantified in flat-mounted retina and flat-mounted choroid perfused with FITC-D. (A) Representative images of flat-mounted choroid and flat-mounted retina. Red arrows indicate NV. (B) Quantification of NV number and area for SRNV and IRNV in the vehicle group (n = 6) and Feno-FA group (n = 7). Data were expressed as mean ± SEM and analyzed by unpaired Student's t-test. **P < 0.01; ***P < 0.001.

We further evaluated the effects of PPARα activation on the formation of SRNV and IRNV in *Vldlr^−/−^* mice. As shown by flat-mounted choroid and retina perfused with FITC-D, numbers of SRNV and IRNV were 11.00 ± 0.89 and 44.33 ± 4.04, respectively, in vehicle-treated *Vldlr^−/−^* mice (*n* = 6), and were decreased to 5.14 ± 1.01 and 24.29 ± 3.09, respectively, in the Feno-FA group (*n* = 7), which represents an approximately 50% reduction for both SRNV and IRNV (Fig. 5). The areas of SRNV and IRNV were also decreased substantially in Feno-FA–treated *Vldlr^−/−^* mice compared with vehicle-treated *Vldlr^−/−^* mice accordingly (Fig. 5). These results suggest that PPARα activation inhibits the development of SRNV and IRNV in *Vldlr^−/−^* mice.

### Fenofibrate Attenuates Retinal Vascular Leukostasis in *Vldlr^−/−^* Mice

**Figure 6:**
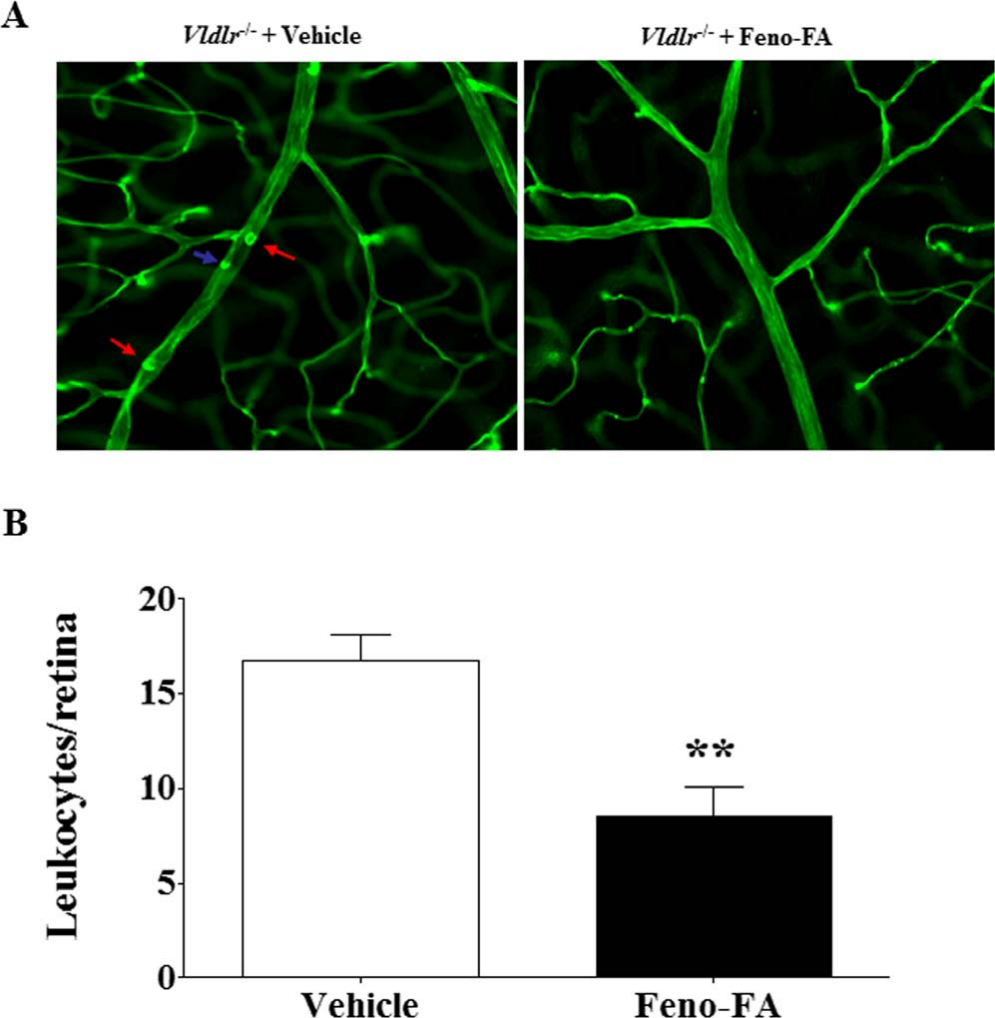
Feno-FA reduced retinal leukostasis in Vldlr^−/−^ mice. Vldlr^−/−^ mice were intraperitoneally injected daily with Feno-FA or vehicle from P13 to P28. At P28, the retinal adherent leukocytes were stained with FITC-conjugated concanavalin-A after the removal of circulating leukocytes. The retinas were then flat-mounted, and adherent leukocytes were visualized by fluorescence microscopy. (A) Representative images of flat-mounted retinas. Arrows indicate adherent leukocytes. (B) Quantification of leukocytes in the vehicle group (n = 7) and Feno-FA group (n = 8). Data were expressed as mean ± SEM and analyzed by unpaired Student's t-test. **P < 0.01.

The effects of PPARα activation on retinal inflammation in *Vldlr^−/−^* mice were evaluated by leukostasis assay. Our previous study reported that retinal vascular adherent leukocytes are increased in *Vldlr^−/−^* mice.^25^ In this study, multiple adherent leukocytes were observed in the retinal vasculature in vehicle-treated *Vldlr^−/−^* mice but fewer adherent leukocytes in Feno-FA–treated *Vldlr^−/−^* mice (Fig. 6A). Quantified data showed that Feno-FA (*n* = 8) significantly decreased adherent leukocytes compared with the vehicle group (*n* = 7; Fig. 6B), suggesting that PPARα activation inhibits the retinal inflammation in *Vldlr^−/−^* mice.

### The Beneficial Effects of Feno-FA on CNV Are PPARα Dependent

**Figure 7:**
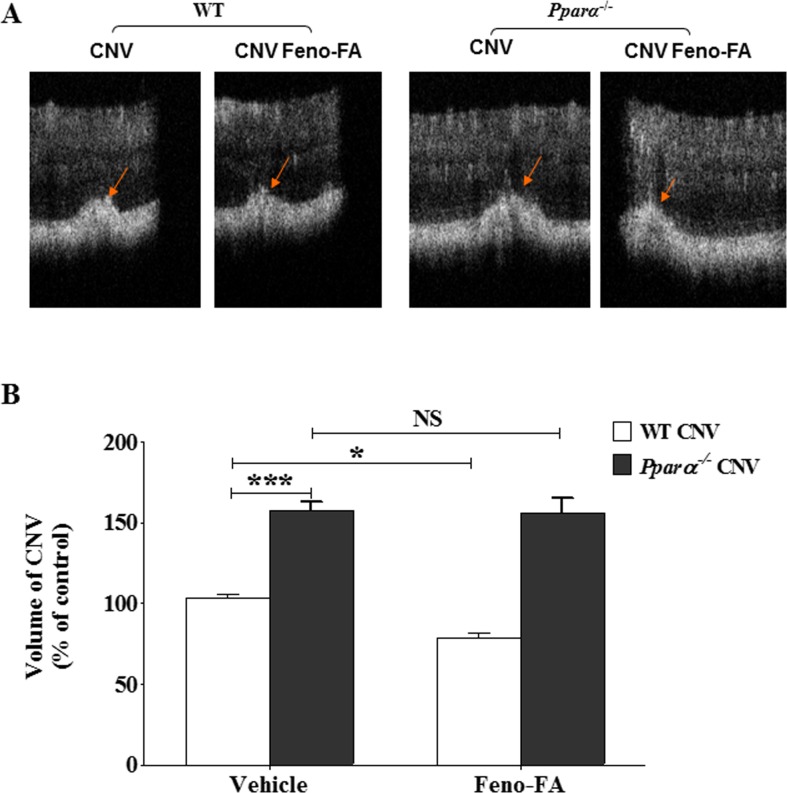
Therapeutic effects of Feno-FA on CNV were PPARα dependent. CNV was induced by laser in age-matched wild-type (WT) and Pparα^−/−^ mice, and then Feno-FA was injected intraperitoneally daily with DMSO as vehicle control for 7 days, beginning at the same day as the laser treatment. CNV volume was quantified using OCT. (A) Representative OCT images showing CNV. (B) Quantification of CNV volumes in WT mice (n = 28), Pparα^−/−^ mice (n = 42), WT mice treated with Feno-FA (n = 16), and Pparα^−/−^ mice treated with Feno-FA (n = 14). Data were expressed as percentages of control and analyzed by 2-way ANOVA followed by Tukey's post hoc tests. *P < 0.05; ***P < 0.001; NS, no statistical significance.

To determine whether the beneficial effects of Feno-FA on CNV occur through a PPARα-dependent mechanism, age-matched WT and *Pparα^−/−^* mice were subjected to laser-induced CNV and then treated with Feno-FA. As shown by CNV volume in OCT (Fig. 7), *Pparα^−/−^* mice (*n* = 42) with laser treatment developed significantly larger sizes of CNV lesions compared with WT mice (*n* = 28), and Feno-FA reduced CNV volume in WT mice (*n* = 16), but not in *Pparα^−/−^* mice (*n* = 14), supporting that Feno-FA's effects on CNV are PPARα dependent.

## Discussion

In this study, we provided evidence that PPARα activation by its agonist, Feno-FA, attenuated retinal NV and SRNV in both laser-induced CNV rats and *Vldlr^−/−^* mice. We also demonstrated that PPARα activation reduced retinal vascular leakage and retinal inflammation in these models. In addition, our results indicated that the beneficial effects of Feno-FA are mediated through a PPARα-dependent mechanism.

Fenofibrate functions as an agonist of PPARα only after it is converted to Feno-FA. In this study, we used Feno-FA to treat the laser-induced CNV model, which recapitulates neovascular AMD phenotypes and is commonly used for studying this disease. Because the CNV progression reaches the peak by approximately 2 weeks after laser photocoagulation in this animal model,^28^ we chose the 2-week postlaser treatment as the endpoint for evaluation. We found that systemic administration of Feno-FA significantly reduces clinically relevant vascular leakage and suppresses formation of CNV in this model, demonstrating an antiangiogenic effect of Feno-FA on laser-induced CNV. Meanwhile, we investigated effects of Feno-FA on retinal angiomatous proliferation (RAP) using *Vldlr^−/−^* mice. RAP is a subtype of neovascular AMD in which the etiologies, molecule mechanisms, and therapy response are different from those of classic CNV.^29–33^ *Vldlr^−/−^* mice, characterized by the presence of SRNV and IRNV, are commonly accepted as a model for RAP.^34–39^ Our results showed that Feno-FA treatment given at P13, when ocular NV is initiated in *Vldlr^−/−^*mice,^37,40^ also inhibits retina vascular leakage and development of SRNV and IRNV in *Vldlr^−/−^*mice. Taken together, these results suggest that Feno-FA has potential to become a clinical intervention for different types of neovascular AMD.

Our study showed that in both the laser-induced CNV rat model and *Vldlr^−/−^* mice, Feno-FA successfully suppressed retinal inflammation, which plays an important role in the pathogenesis of CNV,^26,27^ indicating that its effects on ocular NV in these models can be ascribed, at least in part, to suppression of inflammatory responses, consistent with our previous studies on DR and oxygen-induced retinopathy (OIR) models.^19^ Interestingly, downregulation of VEGF levels in CNV models in the study is less pronounced than that in the OIR model as shown in our previous study.^19^ There are at least two possible reasons for this difference. First, in OIR and CNV models, NV develops from different etiologies and with different molecular mechanisms, and expression of VEGF is mediated by different signaling pathways. It is possible that agonist of PPARα may have different potencies in inhibition of the different signaling pathways to suppress VEGF overexpression in OIR and CNV models. Second, we treated the OIR model with intravitreal injection of fenofibrate (3 μL; 125 μM) in the previous study, but treated the CNV model with intraperitoneal injections with Feno-FA (25 mg/kg/day). The disparities in formulation, dose, and administration route may affect the efficiencies of suppressing VEGF levels in these two models.

As for the mechanism, our study suggests that the beneficial effects of Feno-FA on CNV are through PPARα activation, consistent with our previous study using a DR model.^19^ A recent study, however, reported that fenofibrate inhibits CNV partially through cytochrome P450, a target independent of PPARα.^20^ This disparity may be explained by the following reasons. (1) The study by Gong et al.^20^ used fenofibrate, whereas we used Feno-FA. Fenofibrate, a third-generation fibric acid derivative, is a prodrug, which is hydrolyzed by tissue and plasma esterases to the active metabolite Feno-FA. Fenofibrate is >90% plasma bound, reaches peak concentration in the plasma at approximately 7 hours, and has an elimination half-life of approximately 20 hours.^41^ Feno-FA contains a carboxylic acid moiety but not an isopropyl ester moiety in fenofibrate.^42^ Feno-FA is a PPARα agonist, while fenofibrate is a LXR antagonist.^43^ Fenofibrate may have some off-target effects. It was shown that fenofibrate is a potent inhibitor of cytochrome P450 with affinity >10 times higher than that of PPARα.^44^ Li et al.^45^ reported that fenofibrate inhibits voltage-dependent K^+^ channel (Kv) expression in vascular smooth muscle, while neither another PPARα activator, bezafibrate, affects the Kv current, nor PPARα inhibitor GW6471 changes the inhibitory effect of fenofibrate, indicating that the effect of fenofibrate is independent of PPARα activation.^45^ In addition, fenofibrate suppresses the growth of human hepatocellular carcinoma cells, which was not affected by the PPARα antagonist (GW6471) or by a PPARα-specific siRNA, also suggesting a PPARα-independent mechanism.^46^ Fenofibrate affects retinal endothelial cell survival through the AMPK signal pathway in a PPARα independent manner as well.^47^ (2) We used a lower dose (25 mg/kg/day) of the drug that is similar to the clinical dose in human patients, while Gong et al.^20^ used fenofibrate at 100 mg/kg/day. Fenofibrate at high dose may confer off-target effects. The detailed mechanism for the different observations using *Pparα^−/−^* mice remains to be elucidated.

It has previously been reported that fenofibrate inhibits the expression of proinflammatory and adhesion molecule genes through blocking the activation of NF-κB in endothelial cells.^48^ Our previous study also showed that the anti-inflammatory activity of PPARα is through inhibition of nuclear factor–κB (NF-κB) signaling under diabetic stress.^49^ Thus, it is speculated that Feno-FA activates PPARα and subsequently inhibits NF-κB signaling, leading to attenuation of retinal inflammation in these animal models, which in turn, has beneficial effects on ocular NV.

Anti-VEGF drugs are the principal therapeutic agents for CNV in neovascular AMD.^50^ However, they are not always effective in all of the patients. Fenofibrate may confer beneficial effects on those patients who do not respond to anti-VEGF treatment. Moreover, compared with anti-VEGF compounds, fenofibrate offers some advantages. First, fenofibrate, used for treating dyslipidemia clinically for many years, has proven to be safe and has fewer side effects. Second, fenofibrate can be administrated via the oral route, which is more acceptable to patients and can avoid clinical risks of endophthalmitis or retinal detachment associated with intravitreal injections.^51^ Third, anti-VEGF antibodies are expensive, while fenofibrate is cost-effective, having the potential to reduce the economic burden of patients.

Our study has some strengths. First, we used both models of ocular NV that recapitulate some features of neovascular AMD, including laser-induced CNV rats and *Vldlr^−/−^* mice. Second, both choroidal flat mount and OCT were used for visualizing and quantifying NV size associated with laser-induced CNV. Choroidal flat mount, in which CNV lesions are imaged and quantified in two-dimensional measurements, is the most commonly used technique to evaluate CNV. OCT is a noninvasive technique for imaging the retinal structures in live animals, and the data obtained allowed for precise comparisons of the effects within different treatment groups. Currently, only a few studies have reported application of OCT setups for high-quality, repeatable in vivo imaging of laser-induced CNV.^23,52–54^ Our study was also the first to measure retinal thickness in *Vldlr^−/−^* mice with OCT. Third, this study evaluated antiangiogenic effect of Feno-FA on both IRNV and SRNV in a RAP model.

Despite the contributions of the study discussed here, some questions remain to be addressed. We used a single dose of Feno-FA, and the dose ranges for optimal effects need to be defined. The cellular mechanism and signaling pathways mediating the effect of PPARα on inflammation and angiogenesis need to be elucidated in more detail. Topical administration may be warranted for therapeutic application as well, as not all patients can tolerate oral fenofibrate. Moreover, it is necessary to determine whether the combination of anti-VEGF and Feno-FA has a synergistic effect.

In conclusion, we have demonstrated for the first time that systemic administration of Feno-FA suppresses vascular leakage and formation of ocular NV in models that recapitulate some features of neovascular AMD. This effect might be ascribed to inhibiting inflammatory pathways through a PPARα-dependent mechanism. These results provide the basis for future clinical trials to evaluate the effects of Feno-FA or fenofibrate in human patients with neovascular AMD.

## Supplementary Material

Supplement 1
