# Supplementary material for: Therapeutic Effects of PPARα Agonist on Ocular Neovascularization in Models Recapitulating Neovascular Age-Related Macular Degeneration
Source: Invest Ophthalmol Vis Sci. 2017 Oct;58(12):5065–75. doi: 10.1167/iovs.17-22091 (PMC5633006; doi:10.1167/iovs.17-22091)
Supplement: Supplement 1 [file iovs-58-11-59_s1.pdf]

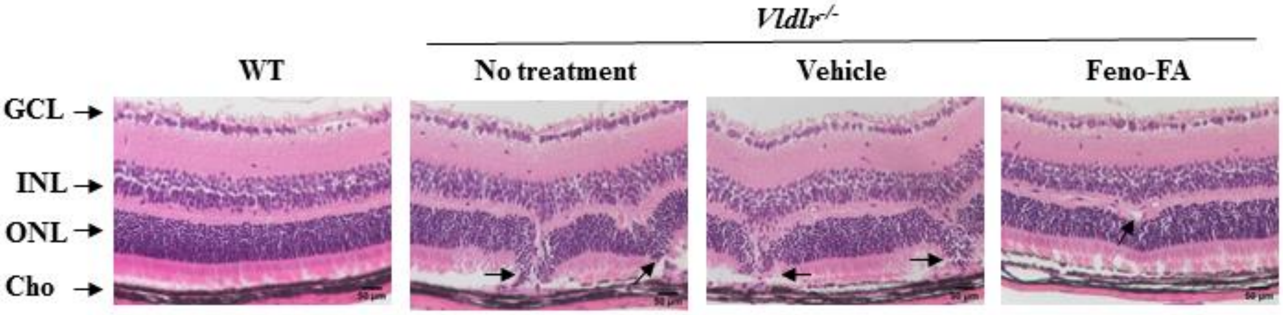

Supplemental Figure 1. H&E-stained retinal sections from age-matched WT mice, *Vldlr*$^{-/-}$ mice, *Vldlr*$^{-/-}$ mice with vehicle treatment, *Vldlr*$^{-/-}$ mice with Feno-FA treatment. *Vldlr*$^{-/-}$ mice were intraperitoneally injected daily with Feno-FA or vehicle from P13 to P28. NV was observed in intra-retina or sub-retina with a disorganized inner nuclear in *Vldlr*$^{-/-}$ mice. Arrows indicated intra-retinal or sub-retinal NV. GCL, ganglion cell layer; INL, inner nuclear layer; ONL, outer nuclear layer; Ch, choroid. Scale bars: 50 µm.
